# A Long-Felt Want in Atlanta

**Published:** 1886-09

**Authors:** 


### A LONG-FELT WANT IN ATLANTA.

A marked feature which distinguishes modern civilization from all the civilizations which have preceded it consists in the attention which is bestowed upon the care of the sick, the maimed and the unfortunate. Greece was famed for her literature and her arts, Rome for the glory of her conquests, yet their beggars and pauper sick died in the streets unpitied. Even among the "chosen people," Lazarus starved at Dives' gate and canine saliva formed the only dressing for his sores. But to-day the establishment and support of eleemosynary institutions are considered as much the duty of government as the maintenance of jails and reformatories. With the advance of every State or community in culture and enlightenment, the number and excellence of such institutions have increased *pari passu.* So true is this that a scale representing the amount of public care bestowed upon the sick in the different parts of the world would be almost identical with a scale representing the relative advance in all that pertains to civilization.

In small communities hospitals are not a necessity. It is only where the collection of a large population into one place has brought with it the necessary accompaniments of extensive railroad connections, large manufactories and varied industries, with their attendant dangers to life and limb, that sudden emergencies and grave accidents require the skilled care which only a hospital can command. It is only in large cities that the pauper element is so great that humanity demands public provision for their comfort and care when sick. Hence a lack of such charities which would be excusable and inevitable in a small community becomes disgraceful and humiliating in one which claims a place among the leading cities of the land.

What, then, shall be said of Atlanta, a city of over fifty thousand inhabitants, the capital of the Empire State of the South, in which eight railroads centre, upon whose air the hum of a thousand diversified industries rises day and night, and yet which has no hospital that is worthy of the name? Are we behind the age in civilization? Are we lacking in humanity? Do we have no railroad disasters? Are we exempt from the ordinary accidents of life? Have we no pauper sick? Which of these reasons will be advanced to explain why we have no hospital facilities which can compare with those of such smaller cities as Macon, Augusta and Savannah?

What Atlanta needs is a hospital constructed upon the most approved plan of modern hospital architecture, capable of accommodating from 75 to 100 patients, furnished with all the appliances requisite for scientific treatment of disease, placed in charge of an able corps of physicians, selected without reference to the disgraceful personal dissensions and petty spites which have so long existed, supported by annual appropriations from the public funds, and governed by a board of trustees who shall be selected from among our most responsible citizens and who shall serve without pay. Not until this is done can any Atlanta man, medical or lay, say that his city has discharged the obligations resting upon her. Not until then can it be claimed that Atlanta is the peer of other cities of equal size.

Next April the Georgia Medical Association will be our guests for three days. The older members of that Association, knowing our failing, will probably, out of consideration for our feelings, avoid the mention of this subject. But some new member, fresh from his studies in the hospitals of Philadelphia and New York, or it may be of Paris and Vienna, after gazing with admiration upon our public and private buildings, of which Atlanta may so well be proud, will turn to us with a look of fond expectancy in his eyes and say, "Now show me your City Hospital." Shall we feel swelling within our hearts the pride which every Atlantian should feel in his native city, as we show him what Atlanta does for her sick poor? No! with downcast eyes, with faces flushed with shame, with laggard and reluctant steps, we must escort him to Waverly Place and there show to him the spacious extent, the wonderful beauty and the generous equipments of the "Benevolent Home."

